# Uncertainty-Aware Explainable AI for Pancreatic Cysts: Identifying Deep Learning Vulnerabilities and Ensuring Safe Clinical Triage in IPMN Management

**DOI:** 10.21203/rs.3.rs-9096790/v1

**Published:** 2026-03-22

**Authors:** Halil Ertugrul Aktas, Gorkem Durak, Andrea Mia Bejar, Ziliang Hong, Rutger Hendrix, Hongyi Pan, Elif Keles, Fergan Bol, Yavuz B Taktak, Cagdas Topel, Yalcin Tur, Zheyuan Zhang, Neil R Chatterjee, Yuri Velichko, Concetto Spampinato, Ivo G Schoots, Marco J Bruno, Chenchan Huang, Tamas Gonda, Alpay Medetalibeyoglu, Gulbiz Dagoglu Kartal, Sukru Mehmet Erturk, Lili Zhao, Candice Bolan, Frank H Miller, Michael B Wallace, Rajesh N Keswani, Ulas Bagci

**Affiliations:** Northwestern University; Northwestern University; Northwestern University; Northwestern University; University of Catania; Northwestern University; Northwestern University; University of Health Sciences Istanbul Bakirkoy Dr. Sadi Konuk Training and Research Hospital; Istanbul University Faculty of Medicine; Northwestern University; Stanford University; Northwestern University; Northwestern University; Northwestern University; University of Catania; Erasmus MC; Erasmus MC; New York University; New York University; Istanbul University Faculty of Medicine; Istanbul University Faculty of Medicine; Istanbul University Faculty of Medicine; Northwestern University; Mayo Clinic; Northwestern University; Mayo Clinic; Northwestern University; Northwestern University

**Keywords:** Radiomics, Deep Learning, IPMN, Pancreatic Cysts, Explainable Artificial Intelligence, Uncertainty Quantification

## Abstract

Despite growing interest in AI for pancreatic cyst management, no prior study has systematically investigated how lesion characteristics influence model behavior or provided a comprehensive explainability analysis in IPMN malignancy risk stratification. We present the first multi-center study integrating explainable AI with uncertainty quantification to evaluate how cyst type, size, and location affect deep learning performance in IPMN malignancy prediction. Our retrospective study analyzed 170 IPMNs from seven centers using a radiomics-deep learning fusion model. Cases were stratified by dysplasia grade, IPMN type, size, and location; with model interpretability assessed using SHAP, LIME, Grad-CAM, and uncertainty quantification. Overall accuracy was 67.1%, with model uncertainty lower for correct versus incorrect predictions (0.72 vs 0.78, p < 0.001). On subgroup analysis: Low-Risk lesion accuracy exceeded High-Risk (81.3% vs. 42.9%, p < 0.001), BD accuracy was inversely correlated with cyst size while MD remained stable, and body and whole-pancreas cysts stratified more accurately than tail cysts (77.8%, 76.6% vs. 47.8%). AI models face significant, previously uncharacterized performance drops when evaluating complex high-risk IPMNs. Our study shows that integrating uncertainty quantification can successfully flag unreliable predictions, enabling a selective-prediction framework where clinicians can confidently rely on AI for low-risk, low-uncertainty lesions while deferring high-uncertainty cases to expert review.

## 1. INTRODUCTION

Intraductal papillary mucinous neoplasms (IPMNs) represent up to 80% of the 3 million pancreatic cysts diagnosed annually in the United States ^[1–5]^, creating a significant healthcare burden with lifetime costs reaching $171,000 per patient^[2, 6]^. These lesions present a clinical dilemma: although at risk of malignant transformation, the majority remain benign, resulting in unnecessary surveillance and interventions ^[4, 5]^. Furthermore, current guidelines fail to reliably distinguish between these outcomes, resulting in a costly paradox of simultaneous overtreatment and missed cancer. Overall, these issues demonstrate that pancreatic cysts are not yet fully understood, highlighting the need for improved clinical management systems ^[6–10]^.

Multiple factors affect clinical evaluation of IPMNs, complicating patient management. Varying malignancy potential between IPMN cyst types (1–38% for branch duct (BD)-IPMNs and 33–85% for main duct (MD)- and mixed-type IPMNs) and user inconsistency in guideline interpretation causes suboptimal patient management^[4, 5, 11, 12]^. This can result in over-management for low-grade lesions unlikely to develop into cancer, exposing patients to avoidable risks and complications. Failing to identify high-risk lesions early can delay necessary intervention, leading to missed opportunities for timely and potentially curative treatment^[4, 9, 13, 14] [15–18]^. Therefore, more objective and advanced risk assessment methods are needed for more accurate and consistent patient management.

The 2024 Kyoto Criteria represent the latest attempt to standardize IPMN management, integrating MRI, endoscopic ultrasound with fine-needle aspiration (EUS-FNA), and molecular analysis ^[4]^. Yet each modality carries fundamental limitations ^[4, 7, 19, 20]^. MRI and magnetic resonance cholangiopancreatography (MRCP) demonstrate inadequate sensitivity for high-grade dysplasia, particularly in small lesions ^[21,22]^. EUS-FNA provides tissue-specific information but involves procedural risks, operator-dependent variability, and cannot confidently exclude malignancy ^[23–25]^. There is an urgent, unmet need for objective, reliable risk stratification tools that can guide clinical decision-making with greater precision than current approaches allow.

Artificial intelligence (AI) offers a promising solution. Radiomics and deep learning (DL) can extract quantitative imaging features beyond human visual perception ^[26]^, and recent studies demonstrate encouraging performance for IPMN classification ^[27–31]^. However, a critical barrier impedes clinical adoption: the “black box” nature of AI predictions renders them fundamentally incompatible with medical decision-making, where clinicians must understand and trust the basis for recommendations that determine patient outcomes. Addressing these challenges, explainable artificial intelligence (XAI) has emerged to fulfill this need by providing insights into model decision-making processes, identifying the specific imaging features that drive predictions. Understanding the confidence of AI predictions enables clinicians to identify scenarios where AI predictions are less reliable and warrant increased awareness of model limitations ^[32–34]^. Yet despite its recognized importance, no prior study has comprehensively applied XAI methods to IPMN risk stratification. Existing pancreatic AI research has focused predominantly on performance metrics, leaving clinicians without insight into when and why models succeed or fail. Equally absent is a systematic investigation of how fundamental IPMN characteristics—type, size, and location—influence model behavior. This knowledge gap represents a major impediment to translating AI promise into clinical practice.

A critical, often unaddressed danger in deploying deep learning for pancreatic cysts is silent failure, instances where an AI model confidently provides an incorrect prediction on a complex lesion. While most literature focuses on maximizing theoretical accuracy on balanced datasets, real-world clinical integration requires knowing exactly when a model is likely to fail. In this study, we deliberately challenge our previously established radiomics-deep learning fusion model with a highly complex, histopathologically-confirmed cohort representing the most diagnostically difficult cases encountered in clinical practice. Rather than presenting AI as an infallible diagnostic replacement, we introduce a comprehensive XAI and Uncertainty Quantification framework designed to act as a ‘safety net’ by identifying AI vulnerabilities across specific Kyoto criteria subgroups and enabling a human-in-the-loop triage system.

## 2. METHODS

### 2.1. Study Design and Patient Population

**Figure 1:**
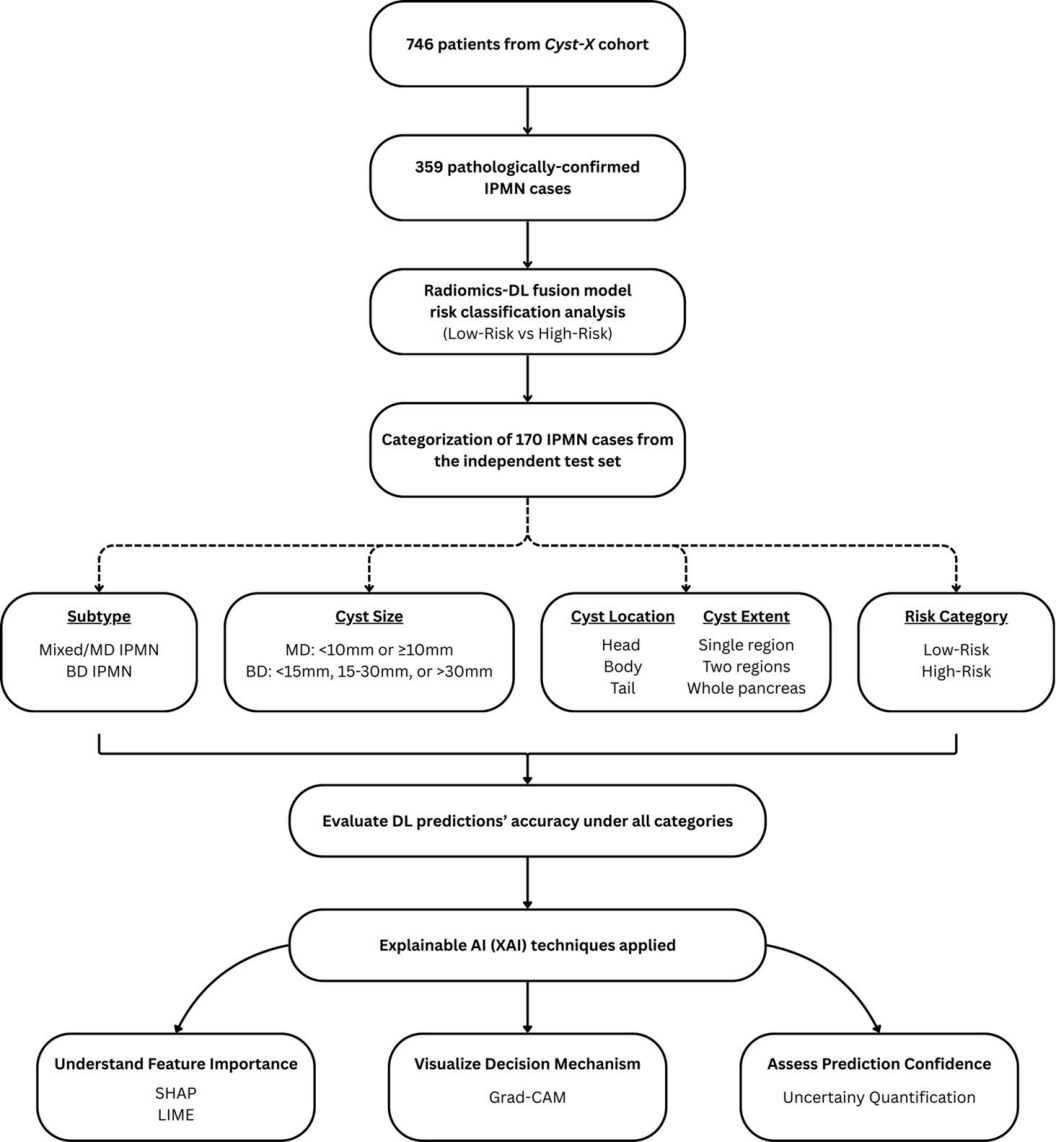
Cohort selection and methodology flowchart.

The imaging dataset was sourced from the Cyst-X dataset, previously established by our collaborative research consortium and made publicly available. This dataset comprises of 746 patients from seven academic medical centers ^[35]^. Of these patients, we included cases with only histopathologically confirmed IPMNs, yielding a cohort of 359 patients that was also applied to our earlier work^[36]^. For this in-depth explainability analysis, we evaluated a subset of 170 cases from the 359-patient cohort, corresponding to the independent test set of our radiomics-DL fusion model (Fig. 1). The following sections outline the categorization framework and analytical methods used in this study.

### 2.2. IPMN Categorization for Performance Analysis

To enable the first systematic analysis of the influence of IPMN characteristics in AI performance, we developed a comprehensive categorization framework encompassing four clinically relevant dimensions: risk group, cyst type, anatomical location, and size based on Kyoto guideline thresholds. These dimensions reflect established clinical risk markers whose relationship to AI performance has never been systematically investigated.

#### 2.2.1 Cyst Type and Malignancy Risk Categorization

Dysplasia grades of BD-IPMNs were determined from pathology results. All MD-IPMN and mixed-type IPMN cases underwent surgical resection with pathological evaluation. Subjects were stratified based on dysplasia grade: lesions exhibiting low-grade dysplasia (LGD) were classified as Low-Risk, while lesions with high-grade dysplasia (HGD) and/or invasive carcinoma (IC) were classified as High-Risk.

#### 2.2.2 Cyst Location Categorization

Lesions were analyzed using both region and extent categorizations. For the region analysis, lesions were classified into six groups: (1) head, (2) body, (3) tail, (4) head + body, (5) body + tail, or (6) whole-pancreas (head + body + tail). For the extent analysis, the same cysts were recategorized based on the extent of pancreatic involvement: confined to a single region, spanning two regions, or involving the whole-pancreas.

This dual framework enabled evaluation of both focal and diffuse cyst involvement patterns, as extensive pancreatic involvement may influence both surgical planning and model prediction accuracy (Table 2).

#### 2.2.3 Cyst Size Categorization

Cyst size categorization was performed using type-specific thresholds informed by clinical guidelines and prior evidence ^[4]^. For BD-IPMNs, maximum cyst diameter was categorized as <15 mm, 15–30 mm, or > 30 mm. The ≥ 30 mm threshold represents a worrisome feature per Kyoto guidelines, while the 15 mm cutoff was supported by a multicenter surveillance study demonstrating that cysts ≥15 mm carried significantly increased risk of developing worrisome features or high-risk stigmata compared with cysts <15 mm, even when initially classified as low-risk BD-IPMNs ^[4, 11]^.

**Figure 2:**
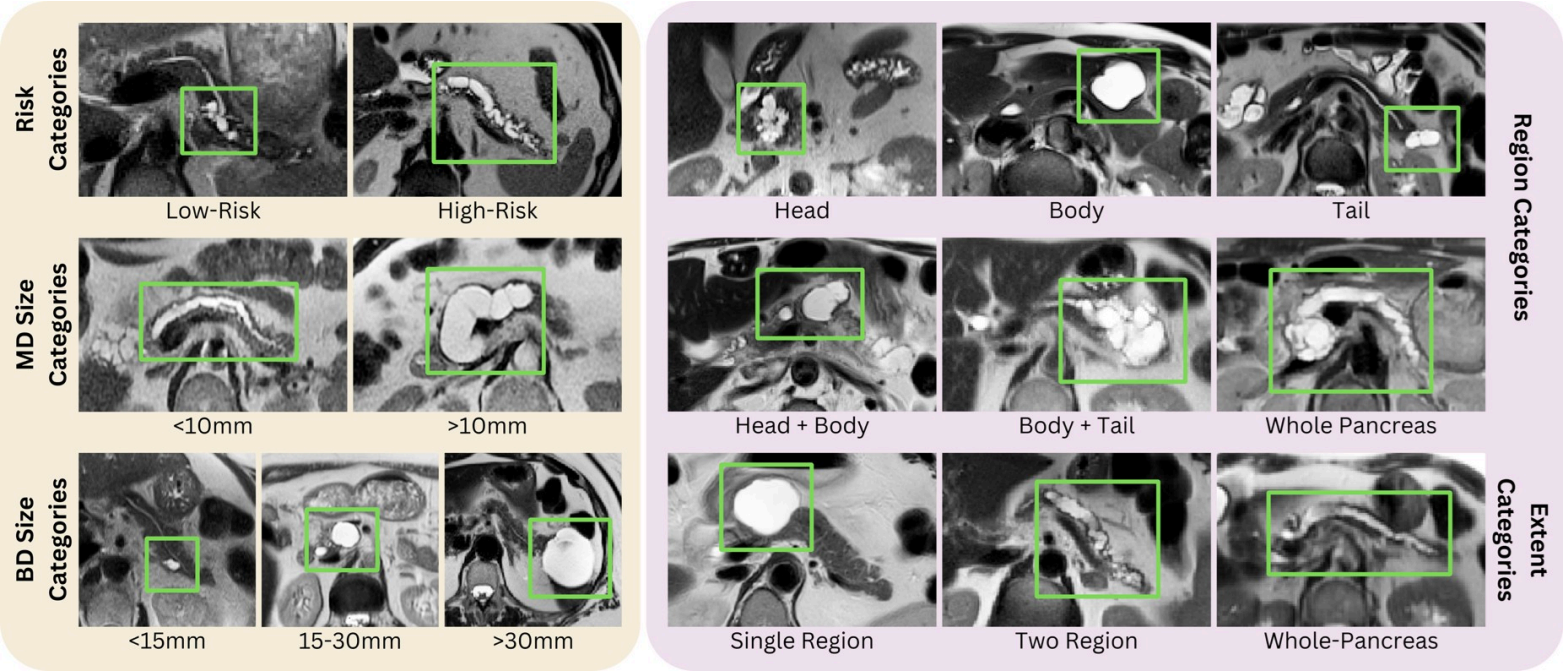
Region Categories Representative cases of in-depth categorization from our cohort. Left panel (yellow) - Risk Categories: (Upper row) Low-risk and High-risk IPMNs; (Middle row) Main Duct (MD) Size Categories: IPMNs with MPD diameter < 10mm versus ≥ 10 mm; (Bottom row) Branch Duct (BD) Size Categories: IPMNs stratified by maximum cyst diameter <15 mm, 15 – 30 mm, and > 30 mm. Right panel (pink) - (Upper row) Region Categories: IPMNs categorized by location, including head, body, tail, head + body, body + tail, and whole-pancreas; (Bottom row) Extent Categories: Classified by cyst distribution as single region, two regions, or whole-pancreas involvement.

For MD-IPMNs, categorization was instead based on main pancreatic duct (MPD) diameter, stratified as <10 mm or ≥10 mm, with the latter representing high-risk stigmata according to the Kyoto guidelines ^[4]^ (Fig. 2).

### 2.3. AI Framework for IPMN Classification

The radiomics–deep learning (DL) fusion model evaluated in this study corresponds to our previously published IPMN risk stratification framework^[36]^, in which model development, performance optimization, and validation were previously performed. The present study focuses on explainability analysis of this established model rather than model development or optimization. Therefore, the model was applied without architectural or training modifications to enable systematic explainability and uncertainty analysis across clinically relevant IPMN subgroups.

#### 2.3.1 Manual Segmentation and Segmentation Quality Assessment

Manual segmentation of each IPMN lesion was performed using ITK-Snap (Version 4.2.0) by an interdisciplinary team with expert radiologists’ review to ensure accuracy and consistency (Fig. S1)^[37]^. For BD-IPMN subjects, the index lesion was defined as the cyst sampled in EUS-FNA or surgically resected. Approximated MD cyst boundaries were discussed among abdominal radiologists and decided by consensus prior to segmentation due to the complex nature of their anatomy. Segmentation quality and reproducibility were validated through interobserver and intraobserver agreement analyses, with complete methodological details provided in our prior publication ^[36]^.

#### 2.3.2 Radiomics-Deep Learning Fusion Algorithm for IPMN Risk Stratification

The data from individual centers were organized into four distinct trial configurations for testing purposes, while the remaining data served as a basis for cross-validation procedures. To replicate real-world clinical scenarios, each trial’s test set comprised complete datasets from one or two centers, resulting in variable test set sizes across trials. Four test groups were divided as Trial 1 (n = 29), Trial 2 (n = 35), Trial 3 (n = 35), and Trial 4 (n = 75). Trial groups were strategically selected to ensure balanced representation of low-risk and high-risk cases across both training and testing datasets.

**Figure 3:**
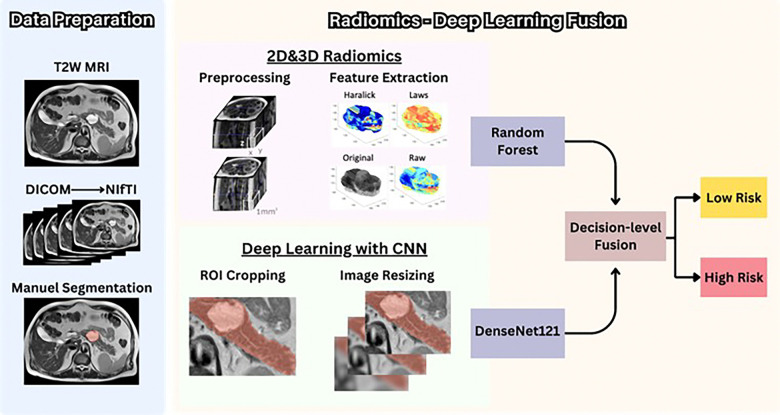
Our radiomics-deep learning fusion algorithm pipeline.

Our hybrid radiomics-DL fusion approach was constructed by integrating probabilities from a radiomics-based Random Forest classifier and a DL based DenseNet121 model^[38] [39]^(Fig. 3). Radiomic feature selection used 5-fold cross-validation, retaining features with Spearman correlation coefficients below 0.6 to reduce redundancy. Both the Random Forest radiomics model and the DL architecture were retrained on identical training datasets, with consistent data partitioning to ensure valid comparisons. After model training, the probability outputs from both approaches were combined to form an ensemble, which was assessed on the validation and test datasets.

The decision-level fusion strategy combined probabilities from both DenseNet121 and Random Forest models. Building upon our previous methodology ^[40]^, we implemented an exact sampling approach for the fusion technique and identified optimal hyperparameters through grid search within a five-fold cross-validation framework. The fusion algorithm incorporated two key hyperparameters: threshold t and weight k. When radiomics predictions surpassed threshold t, the final output relied exclusively on the radiomics prediction, effectively discarding the DL prediction. Conversely, when the threshold is not exceeded, the fusion output represents a weighted combination of both model predictions, with the radiomics model weight set to 1−k and the DL model weight assigned as k.

**Figure 4:**
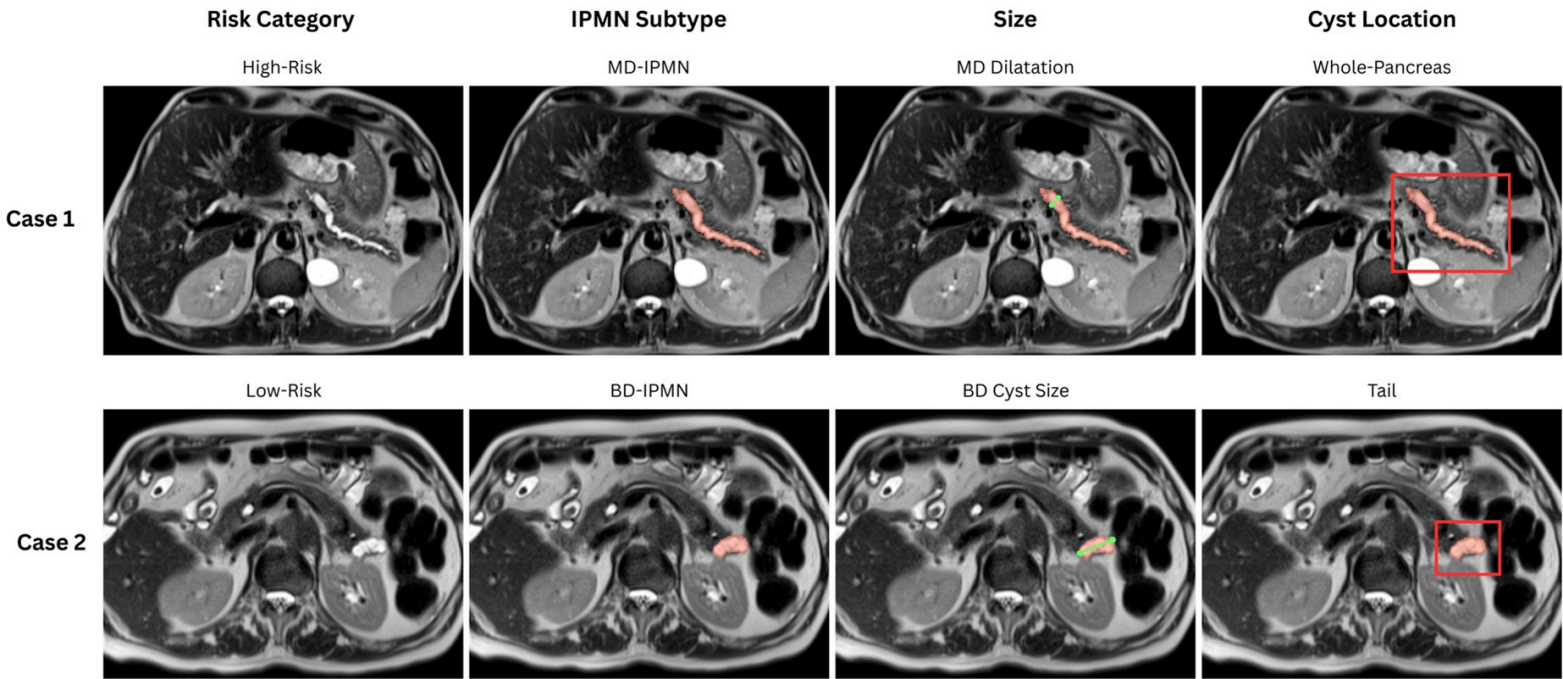
Representative IPMN characterization by risk category, type, size, and location. Axial T2-weighted MRI images demonstrating the classification framework applied to IPMN cases. Case 1 shows a High-Risk MD-IPMN with main duct dilatation and whole-pancreas involvement. Case 2 demonstrates a Low-Risk BD-IPMN with a cyst in the pancreatic tail. Red overlays indicate segmented regions of interest. Each case illustrates the four-dimensional characterization approach: **(1)** risk stratification based on dysplasia grade, **(2)** type classification (MD-IPMN vs BD-IPMN), **(3)** size measurements (main duct diameter vs cyst diameter), and **(4)** anatomical location mapping.

This fusion methodology was executed twice, utilizing either 2D or 3D radiomic feature sets. Weighted averaging was implemented to account for varying subject representation across participating centers. Our complete fusion workflow is illustrated in Fig. 4.

### 2.4 Explainability Frameworks

To elucidate the model’s decision-making process and identify key imaging biomarkers, we performed comprehensive explainability analyses using SHapley Additive exPlanations (SHAP) and Local Interpretable Model-agnostic Explanations (LIME) for feature attribution, Grad-CAM for spatial visualization, and uncertainty quantification for confidence estimation across the entire cohort.

#### 2.4.1 SHAP and LIME analysis

We implemented two complementary explainability methods-SHAP and LIME-to quantify how individual radiomic features contributed to model predictions ^[41,42]^.

SHAP assigns to each radiomic feature a contribution score by computing its influence across all possible feature combinations. This provides both global feature importance (identifying which features are most influential across all cases) and local interpretability (showing how features contribute to individual predictions). SHAP values were computed for all samples in each test set using the TreeExplainer, with the model output set to probability mode to explain predicted class probabilities. Global feature importance was derived by aggregating SHAP values across all samples, while local SHAP values indicated the direction and magnitude of each feature’s contribution to individual high-risk predictions. To assess explanation strength, we computed the total absolute SHAP value for each prediction by summing the absolute values of all feature contributions.

LIME generates case-specific explanations by systematically perturbing radiomic features and observing how predictions change, then fitting a simplified linear model to approximate the complex model’s behavior locally. We used LimeTabularExplainer with approximately 5,000 perturbations per case. Total absolute LIME values were similarly computed by summing absolute feature contributions. While SHAP provides globally consistent attributions across the entire dataset, LIME is optimized for individual case interpretation. These methods enabled us to identify which imaging characteristics (e.g., texture patterns, shape descriptors) drove the model’s risk predictions and to evaluate whether explanation strength correlates with prediction confidence.

#### 2.4.2 Grad-CAM

Grad-CAM was used to visualize the deep learning model’s inference process. Grad-CAM computes the gradient of the network’s output with respect to the feature maps in the last convolutional layer^[43]^. These gradients are then used to produce a heatmap that highlights the image regions most relevant to the model’s decision. In our study, Grad-CAM enabled us to generate attention maps that revealed whether the model focused on the actual cyst or on surrounding tissues during classification (Fig. S2).

#### 2.4.3 Uncertainty Quantification

For the radiomics-fused DL method, we implemented and trained our models using Monte Carlo (MC) dropout with a dropout probability of 0.3. At inference time, we performed $T_{pass}$ stochastic forward passes $\left(T_{pass}=30\right)$ to approximate the predictive distribution and estimate uncertainty ^[44]^.

In our two-branch uncertainty estimation architecture, we further leveraged the principle that the radiomic features random forest classifier inherently quantifies uncertainty through ensemble voting ^[38]^. Each of the $T_{pass}$ forward passes from the deep learning branch was paired with predictions from all 47 decision trees of the random forest, resulting in a combined set of T=30×47 predictive samples. This extended distribution enabled a robust estimation of both data- and model-driven uncertainties.

The SoftMax outputs from these passes were aggregated to compute the mean predictive probability $\hat{p}=\frac{1}{T}\sum_{t=1}^{T}p_{t}$, where $p_{t}\in R^{C}$ is the class probability vector at pass t and C is the number of classes. The total predictive uncertainty was computed using Shannon entropy of the mean prediction given by $H(\hat{p})=-\sum_{c=1}^{C}{\hat{p}}_{c}log\left({\hat{p}}_{c}+\epsilon \right)$, where ϵ is a small constant added for numerical stability. Aleatoric uncertainty was estimated by computing the expected entropy across the T samples, expressed as $E[H(p)]=\frac{1}{T}\sum_{t=1}^{T}\left(-\sum_{c=1}^{C}p_{t,c}log\left(p_{t,c}+\epsilon \right)\right)$. Epistemic uncertainty, reflecting model confidence, was calculated as the mutual information between the predictions and the model posterior, given by the difference $I=H(\hat{p})-E[H(p)]$^[44, 45]^. To facilitate comparison across tasks with different numbers of classes, each of these uncertainty measures was normalized by the maximum possible entropy log(C), resulting in normalized total, aleatoric, and epistemic uncertainty scores. This framework enabled a systematic, interpretable assessment of model confidence on this classification task.

### 2.5 Statistical Analysis

Model performance was evaluated using standard classification metrics. The area under the receiver operating characteristic curve (AUC) was calculated to assess discrimination ability. Sensitivity and specificity were computed at the optimal operating point determined by Youden’s index. The Brier score was calculated to evaluate overall prediction accuracy and calibration, with lower values indicating better performance.

Comparisons between categories and predictions (correct and incorrect) were evaluated using Fisher’s exact tests. Associations between categories and prediction uncertainty (a continuous variable) were assessed using Analysis of Variance (ANOVA) models, with pairwise p-values adjusted using the Tukey method for feature variables with more than 2 categories.

To evaluate the relationship between explanation strength and prediction confidence, associations between total absolute SHAP/LIME values and model uncertainty were analyzed using Spearman rank and Pearson correlation analyses. Scatter plots with linear regression lines and 95% confidence intervals were used to visualize these relationships on a per-case basis. Statistical significance is defined as p< 0.05. Analyses were conducted using R, SciPy, Statsmodels, Scikit-learn, SHAP, and LIME libraries.

## 3. RESULTS

### 3.1. Cohort Characteristics

The cohort was stratified by pathologically confirmed dysplasia grade into Low-Risk (low-grade dysplasia) and High-Risk (high-grade dysplasia or invasive carcinoma) groups. The Low-Risk group consisted of 107 scans, comprising 74 BD-IPMN cases (79.6% of 93 total BD-IPMN cases) and 33 MD or mixed-type IPMN cases (42.9% of 77 total MD/mixed-type cases). The High-Risk group included 63 subjects: 19 with BD-IPMN (20.4% of all BD-IPMN cases) and 44 with MD/mixed-type IPMN (57.1% of all MD/mixed-type cases). (Table 1A).

In the BD-IPMN group, mean maximum cyst diameter was 21.9 mm (SD = 11.7, median = 18.2 mm, IQR 14.0–29.0 mm) in Low-Risk cases and 28.8 mm (SD = 15.4, median = 25.1 mm, IQR 15.5–37.5 mm) in High-Risk cases. In MD-IPMNs, the mean MPD diameter was 9.7 mm (SD = 5.0, median = 8.6 mm, IQR 6.8–9.9 mm) for Low-Risk cases and 11.8 mm (SD = 5.8, median = 10.3 mm, IQR 7.9–14.1 mm) for High-Risk cases. These size categories were used to evaluate model performance across different cyst dimensions, as reported in Table 1A. (Fig. 4).

Region analysis showed Low-Risk lesions (n = 107) most frequently isolated to the head (37.4%), while High-Risk lesions (n = 63) predominantly showed whole-pancreas involvement (41.3%). Extent analysis showed Low-Risk lesions were more often confined to a single region (65.4%), while High-Risk lesions more frequently involved the whole-pancreas (41.3%). Detailed distributions are provided in Table 1B.

### 3.2. Model Performance Overview

We evaluated the performance of the radiomics-deep learning fusion model for malignancy risk prediction across IPMN characteristics, examining both prediction accuracy and uncertainty as complementary metrics (Table 4). The model achieved an overall accuracy of 67.1% (114/170 cases) in classifying IPMN risk. Performance and uncertainty varied substantially across IPMN subgroups when analyzed by cyst type, size, risk category, and anatomical location.

Model uncertainty was strongly correlated with prediction accuracy. Correct predictions exhibited significantly lower uncertainty (0.72 ± 0.01 s.e.m.) compared to incorrect predictions (0.78 ± 0.01 s.e.m., p<0.0001).

#### 3.2.1 Cyst Types and Risk Analysis

BD-IPMNs demonstrated higher accuracy than MD-IPMNs (71.0% vs 62.3%, p = 0.254), though the difference was not statistically significant. However, the model exhibited significantly lower uncertainty for BD lesions compared to MD lesions (0.72 ± 0.01 s.e.m. vs 0.76 ± 0.01 s.e.m., p = 0.012).

The model’s ability to correctly classify cases differed significantly between risk groups. Low-Risk lesions classified more accurately (81.3%, 87/107) compared to High-Risk lesions (42.9%, 27/63; p < 0.001). Model uncertainty was significantly lower in Low-Risk lesions compared to High-Risk lesions (0.73 ± 0.01 vs 0.76 ± 0.02, p = 0.034).

**Figure 5:**
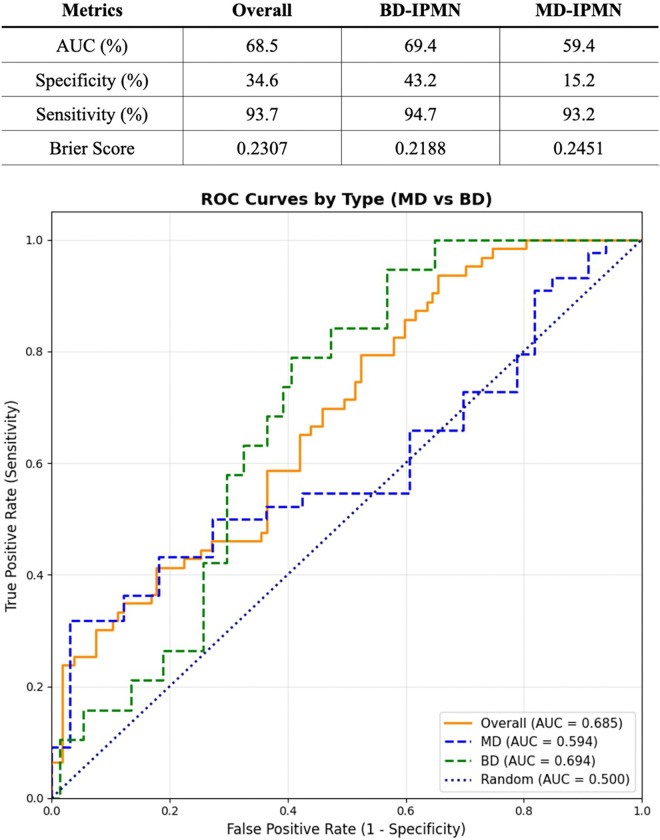
Classification performance metrics and ROC curves stratified by IPMN cyst type. The table summarizes AUC, sensitivity, specificity, and Brier Score for the overall cohort, BD-IPMNs, and MD-IPMNs. ROC curves demonstrate risk stratification performance for BD-IPMNs (green dashed, AUC = 0.694), MD-IPMNs (blue dashed, AUC = 0.594), and the overall cohort (orange solid, AUC = 0.685). The diagonal dotted line indicates random classification (AUC = 0.500).

To evaluate classification performance across cyst subtypes, ROC analysis was performed for the entire cohort and stratified by cyst type, showing moderate overall performance with an AUC of 0.685. Performance differs by subtype, with better discrimination and calibration in BD cysts (AUC = 0.694, lower Brier score) compared with MD cysts (AUC = 0.594), while maintaining high sensitivity across all groups (Fig. 5).

#### 3.2.2 Cyst Size Analysis

For BD-IPMNs, model accuracy declined progressively with increasing cyst size: < 15 mm (81.3%), 15–30 mm (63.2%), and > 30 mm (61.5%); p = 0.164. Furthermore, uncertainty increased significantly with large (> 30 mm) cysts compared to small (< 15 mm) cysts. (0.75 ± 0.02 vs 0.69 ± 0.02, p = 0.048).

In MD-IPMNs, model performance remained stable across ductal diameter categories (<10 mm: 65.9%, ≥ 10 mm: 63.6%; p = 1.000), and uncertainty did not differ significantly between the two groups (p = 0.461).

#### 3.2.3 Cyst Region and Extent Analysis

Model performance varied both by region of the cyst and extent of pancreatic involvement. In the region analysis, accuracy was highest for cysts in the pancreatic body (77.8%), followed by head (69.2%), and lowest in the tail (47.8%). Cases with whole-pancreas involvement achieved 76.6% accuracy, though overall differences among anatomical sites were not statistically significant (p = 0.074). Furthermore, uncertainty showed no significant variation among specific anatomical locations (p = 0.152).

In the extent analysis, accuracy varied across involvement patterns: single region (65.6%), two regions (56.7%), and whole-pancreas (76.6%; p = 0.168). Model uncertainty showed a trend toward higher values with greater pancreatic involvement (single region: 0.73 ± 0.01, two regions: 0.75 ± 0.02, whole-pancreas: 0.76 ± 0.01), though differences were not statistically significant (p = 0.121).

### 3.3. Explainability Analysis

#### 3.3.1 SHAP and LIME Analysis

Across the cohort, model predictions were predominantly influenced by radiomic texture features, particularly entropy- and correlation-based descriptors, which consistently exhibited larger attribution magnitudes across independent test sets. SHAP summary plots demonstrated stable feature importance patterns, with several texture-derived features ranking among the most influential predictors across all evaluation groups (Fig. S3).

Complementary LIME analyses were performed to evaluate local feature contributions using both signed and absolute aggregation strategies. While signed aggregation reflected the average directional influence of features, absolute aggregation captured overall contribution magnitude irrespective of direction. These analyses revealed that several features exerted substantial influence on model predictions but with variable directional effects across samples, suggesting local instability in feature contributions (Fig. S4).

We further examined the relationship between explanation strength and predictive uncertainty. Total absolute SHAP values demonstrated a moderate inverse correlation with uncertainty (Spearman ρ = −0.41, p < 0.001; Pearson r = − 0.38, p < 0.001), indicating that stronger feature attributions were associated with lower uncertainty. In contrast, total absolute LIME values showed no meaningful association with uncertainty (Spearman ρ ≈ 0, p> 0.9; Pearson r ≈ 0, p> 0.6) (Fig. S5). Linear regression with 95% confidence intervals confirmed a statistically significant negative relationship between SHAP magnitude and uncertainty, whereas LIME explanations remained largely uncorrelated across the full range of prediction confidence.

#### 3.3.2 Uncertainty Quantification Analysis

In our cohort, uncertainty differed significantly across key clinical comparisons. Higher uncertainty was consistently associated with incorrect predictions, high-risk lesions, and MD-IPMNs, whereas no significant differences were observed across cyst location or extent of pancreatic involvement. Detailed subgroup analyses are presented in the corresponding sections, with summary statistics provided in Table 2.

To evaluate the clinical utility of uncertainty estimation, we analyzed how prediction accuracy changes when the model defers high-uncertainty cases to human review. We examined three uncertainty types across all cases: combined uncertainty (sum of aleatoric and epistemic components), aleatoric uncertainty (reflecting inherent ambiguity in imaging data), and epistemic uncertainty (reflecting model knowledge limitations).

**Figure 6:**
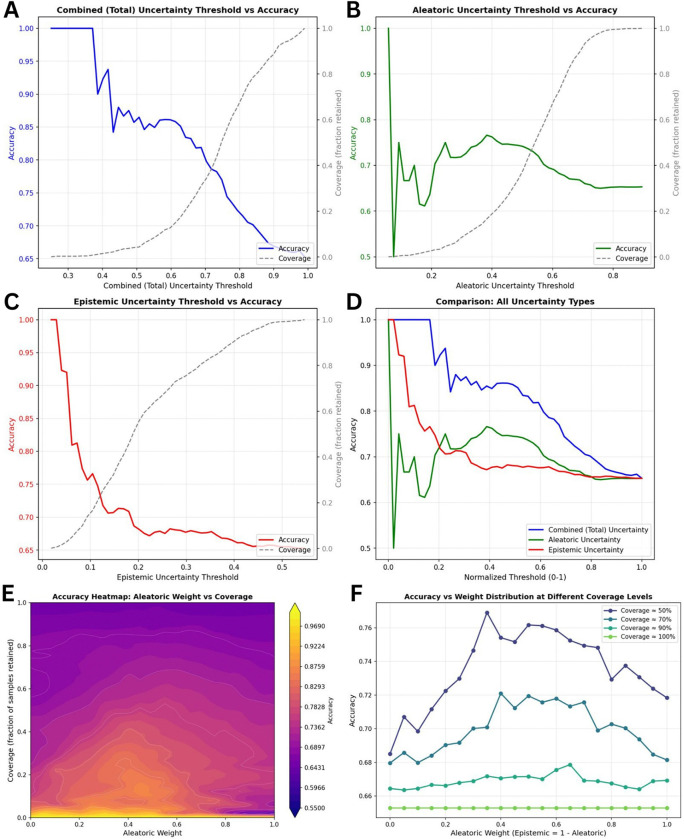
Uncertainty thresholding and optimal weighting of aleatoric and epistemic uncertainty components. **(A-C)** Relationship between uncertainty thresholds and prediction accuracy for combined (blue), aleatoric (green), and epistemic (red) uncertainty, respectively. Accuracy is evaluated as predictions exceeding a given uncertainty threshold are excluded, while dashed curves indicate the corresponding coverage (fraction of retained samples). **(D)** Comparison of all uncertainty types on a normalized threshold scale. **(E)** Accuracy heatmap showing model performance across different weighting schemes between aleatoric and epistemic uncertainty components and varying coverage levels, with brighter colors indicating higher predictive accuracy. **(F)** Accuracy curves at fixed coverage levels illustrating how optimal weighting between aleatoric and epistemic uncertainty varies depending on the desired coverage target.

The relationship between uncertainty thresholds and prediction accuracy when implementing selective prediction strategies is demonstrated in Fig. 6. When the model restricts predictions to only its most confident cases (low uncertainty threshold), accuracy approaches 100%, but coverage-the proportion of cases receiving automated predictions-decreases substantially. Combined uncertainty (total uncertainty) provided the most reliable and consistent tradeoff between accuracy and coverage across all threshold levels. Epistemic uncertainty performed well at identifying the most confident predictions but exhibited instability at higher coverage levels. Aleatoric uncertainty showed the most variable behavior, suggesting limited utility as a standalone criterion for clinical deferral decisions.

To determine whether a weighted combination of aleatoric and epistemic uncertainty could outperform their simple sum, we systematically evaluated different weighting schemes across multiple coverage levels (Fig. 6). At 50% coverage, where half of cases are deferred to radiologists, the optimal weighting assigned 45% to aleatoric and 55% to epistemic uncertainty, achieving 78.3% accuracy compared to 77.3% with equal weighting-a 1.0 percentage point improvement. At 70% coverage, shifting the balance to 60% aleatoric and 40% epistemic weighting yielded 73.0% accuracy. These findings indicate that epistemic uncertainty is more informative when selecting the most reliable predictions (lower coverage), while aleatoric uncertainty becomes relatively more important when accepting a larger proportion of cases (higher coverage). At near-complete coverage (> 90%), the choice of weighting had minimal impact on accuracy, as most cases were included regardless of their uncertainty profiles.

Crucially, the model’s lowest accuracy was observed in High-Risk lesions (42.9%). However, this critical performance gap was successfully captured by our uncertainty metrics. Epistemic uncertainty was significantly higher for these incorrect, high-risk predictions compared to low-risk predictions (p = 0.034). This demonstrates that the algorithm ‘knows what it does not know’. By implementing the combined uncertainty thresholding strategy shown in Fig. 6, we simulated a clinical triage scenario. When the model deferred the top 30–40% of highly uncertain cases to a human radiologist, the automated accuracy on the remaining autonomous predictions increased significantly to approximately 80–85%. This proves that uncertainty quantification can transform a moderately accurate standalone model into a highly reliable triage partner.

## 4. DISCUSSION

Our study presents the first comprehensive explainability evaluation of an AI model for IPMN malignancy risk assessment, analyzing the behavior of our previously published radiomics-DL fusion model^[36]^ across 170 histopathologically confirmed IPMN cases from seven international centers. Using advanced explainability frameworks, including SHAP, LIME, Grad-CAM, and uncertainty quantification, we evaluated how model behavior and prediction reliability vary across clinically relevant IPMN characteristics. The model achieved an overall accuracy of 67.1% on the independent test set. Model uncertainty was significantly lower for correct predictions compared to incorrect predictions (0.72 versus 0.78, p < 0.001), suggesting that uncertainty effectively reflects prediction confidence and highlights its role as a reliability measure for clinical interpretability and trustworthiness. Detailed radiological findings and explainability results are discussed below.

It is important to highlight that sensitivity is much more critical for MRI-based diagnosis, as this is usually followed by a second-line EUS-FNA for confirmation. Since EUS-FNA is considerably more specific, especially when combined with molecular classification, it is probable that the combination of the MRI-Cyst XAI algorithm and EUS will be more accurate. Furthermore, our model’s 67.1% accuracy should be interpreted within the context of our study population. All cases underwent pathological confirmation by surgery or EUS-FNA, representing the challenging subset of patients in whom clinical suspicion warranted invasive procedures. This cohort deliberately excludes the substantial proportion of small, low-risk cysts, which are typically managed with surveillance alone, and lesions with straightforward imaging characteristics that would likely be classified more accurately. Our dataset, therefore, reflects the diagnostically difficult cases in which even clinical guidelines and expert radiologists face challenges, rather than the easier cases that dominate routine practice. In an unselected population including conservatively managed cysts, model performance would likely be higher, as the model would encounter a more favorable case mix reflecting real-world prevalence.

Our algorithm achieved higher classification accuracy for Low-Risk cases (81.3%) compared to High-Risk cases (42.9%; p < 0.001), along with significantly lower prediction uncertainty in Low-Risk cases (p = 0.034). A similar pattern was seen for cyst types, with lower uncertainty for BD-IPMNs than for MD-IPMNs (p = 0.012). This may be because low-risk and BD-IPMN cases tend to have simpler morphology and imaging features, such as uniformity and well-defined borders, making them easier to evaluate. Conversely, high-risk and MD-IPMN cases are more heterogeneous and complex on imaging, which can be more difficult even for expert radiologists. Moreover, even the current reference guidelines demonstrate suboptimal performance in accurately identifying malignancy potential in High-Risk lesions. Despite these, the reduced uncertainty and improved reliability observed in Low-Risk lesions and BD-IPMNs offer clinical value, as confident identification of low-risk conditions can help avoid unnecessary invasive procedures.

Our SHAP analysis consistently identified entropy and correlation-based texture descriptors as the primary drivers of model predictions. From a radiological perspective, high entropy corresponds to increased intra-lesional heterogeneity on T2W MRI. Clinically, this aligns with the biological progression of IPMNs; as low-grade dysplasia transitions to high-grade dysplasia or invasive carcinoma, the cyst fluid and architecture become increasingly complex due to the presence of solid components, mucin density and plugging, or mural nodules. The AI model is effectively mathematically quantifying this visual heterogeneity. However, as our uncertainty analysis shows, when this heterogeneity reaches the extreme levels seen in advanced MD-IPMNs, the imaging data becomes inherently ambiguous (aleatoric uncertainty), prompting the model to correctly signal for expert human intervention.

The model evaluated in this study reflects the intrinsic behavior of our previously published framework, enabling explainability and uncertainty analyses to identify where predictions are reliable and where limitations arise. Building on this, size-based analysis revealed that MPD dilation ≥10 mm is a well-established high-risk feature and is cited in the Kyoto guidelines as one of the high-risk stigmata of IPMNs. While analyzing performance by cyst size, we found that algorithm accuracy remained stable across size categories for MD-IPMN, with 65.9% in the <10 mm category and 63.6% in the ≥10 mm category (p = 1.000). Similarly, for BD-IPMNs, although a trend toward decreasing accuracy with increasing cyst size was observed: <15 mm (81.3%), 15–30 mm (63.2%), and > 30 mm (61.5%), model accuracy showed no statistically significant difference between size groups (p = 0.164). Therefore, the algorithm aligns with the threshold specified in the Kyoto guideline and maintains stable performance across both below- and above-threshold groups. In such scenarios, AI has the potential to assist in confirming or refining risk feature thresholds and to support future clinical trials.

Beyond descriptive performance metrics, our uncertainty analysis demonstrates a clinically actionable strategy for improving the reliability of Al-assisted IPMN risk stratification. By applying predefined uncertainty thresholds, the model can selectively restrict automated predictions to cases in which it is most confident, while deferring higher-uncertainty cases to radiologist review. Under this selective prediction framework, model accuracy increased to approximately 80% at moderate coverage levels, illustrating that uncertainty-aware triage can substantially improve predictive reliability without excluding all automated assistance. Importantly, cases associated with higher uncertainty-such as high-risk lesions and MD-IPMNs-are the same scenarios in which clinical decision-making is most challenging. This underscores the value of uncertainty as an objective signal for heightened human oversight. Rather than replacing clinical judgment, this uncertainty-guided approach supports a hybrid decision model that offers a pragmatic pathway toward safer, more trustworthy integration of AI into pancreatic cyst management.

Explainability analysis demonstrated that prediction uncertainty was inversely associated with SHAP explanation strength, whereas no meaningful relationship was observed with LIME’s. Lower-uncertainty predictions exhibited stronger SHAP attributions, consistent with more confident and coherent feature contributions, while LIME explanations remained relatively stable across the uncertainty spectrum. This divergence likely stems from methodological differences: LIME focuses on local linear approximations optimized for individual predictions, potentially missing global relationships, while SHAP provides globally consistent attributions that better reflect how imaging features influence confident predictions. From a clinical perspective, SHAP explanations may therefore provide added value by informing both interpretability and prediction reliability, helping radiologists identify cases that warrant greater scrutiny.

Similar explainability approaches to those applied in our study are utilized to understand AI classification algorithms in diverse medical applications. To our knowledge, only one study has directly employed these XAI methods to classify pancreatic cystic lesions. Huang et al. (2025) ^[46]^ developed a Multimodal Deep Forest (MDF) model to classify pancreatic cystic lesions into four categories: benign IPMN, malignant IPMN, serous cystadenoma, or mucinous cystic neoplasm. Their model utilized cyst features (e.g., main PD width, cyst communication with main PD, and cyst location) that were manually measured by radiologists from CT (n = 568) and MRI (n = 449) scans, in addition to clinical features (e.g., medical history, blood test results, age, and gender). Additionally, they used SHAP to identify the impact of each feature on the model’s performance. The MDF model achieved an average accuracy of 91.2% and identified the following as the most important predictors: complicated with pancreatitis, main pancreatic duct width, age, communication with the pancreatic duct, CA19–9 level, and CA125 level. Their results demonstrate the value of integrating clinical features with analyses of imaging features. Compared to theirs, our study inputs MRI images directly into the algorithm, enabling us to extract radiomic features and use DL to predict IPMN dysplasia grade. Although their MDF model performed well, manually extracting imaging features limits the number and types of usable features, potentially excluding those that cannot be directly observed by the human eye, and limits the model’s ability to be fully explained. Furthermore, our study focused solely on IPMN and classified lesions by type, size, and location. This is particularly valuable when considering type features that can influence model performance, as MD-IPMN is a higher-risk condition; thus, a combined evaluation of MD and BD IPMN could bias algorithm performance. Our study also collected data from seven international centers, thereby increasing the generalizability of our findings compared to Huang (2025).

A few additional studies have used XAI tools to evaluate pancreatic cancer risk in medical imaging. Pan et al. (2025) and Salanitri et al. (2022) both applied Grad-CAM to generate attention maps of their models’ analysis of MRI images ^[29, 35]^. Heatmaps provide valuable insights into the areas of an image that carry more weight in the model’s decision-making, but they do not identify the specific features that attract the model’s focus. Both studies included Grad-CAM heatmaps as part of their analyses, noting that extra-cystic features in the image were identified but not investigating what these features specifically were. Other studies analyzed the features used by their models to diagnose tumor marker expression with SHAP. These studies either used patient clinical data, urine sample information, or genetic markers as inputs. They successfully identified specific markers that affected their models’ performance and created SHAP-based scatter plots to illustrate the impact of each feature ^[47, 48]^. However, these studies differ in scope from our work, and no other research applies explainable AI techniques as comprehensively as we do, directly addressing the gap faced by clinical AI.

Several limitations should be considered when interpreting our findings. First, the retrospective study design limits causal inference and may introduce selection bias when collecting historical data. Second, data gathered over two decades inherently vary in image quality and acquisition technique. Moreover, the relationship between radiomics features and clinical outcomes remains only partially understood, limiting our ability to confirm whether the model focuses on clinically relevant imaging biomarkers. Since these post-hoc explanations may not fully capture the complex internal workings of deep neural networks, and the data are limited for definitive conclusions, further research is needed to clarify the differences in accuracy. Despite these limitations, our study offers new insights into AI model behavior across various IPMN characteristics and underscores the potential of uncertainty metrics to enhance clinical interpretability.

## CONCLUSION

This study presents the first comprehensive explainability analysis for AI-based IPMN risk stratification, directly addressing the critical transparency gap that has impeded clinical adoption of pancreatic AI. Our systematic investigation establishes several novel findings: IPMN characteristics profoundly influence AI performance; uncertainty quantification enables identification of unreliable predictions; and explanation strength correlates with prediction reliability. These findings address an urgent unmet need in pancreatic cyst management. As AI systems move toward clinical deployment, the framework established here-comprehensive explainability integrated with uncertainty quantification-provides the transparency necessary for safe, effective human-AI collaboration. Rather than replacing clinical expertise, explainable AI can augment it: supporting confident decisions where appropriate and directing attention where clinical judgment remains essential. Our work establishes a new standard for trustworthy, interpretable AI in pancreatic disease and offers a template for clinical AI development across medical imaging.

## Supplementary Material

This is a list of supplementary files associated with this preprint. Click to download.

- XAIIPMNv6supplementary.docx

## Figures and Tables

**Table 1 A.** Average cyst size by IPMN types and risk groups in our cohort.

IPMN Type	Risk Group	n (%)	Mean Size ± SD (mm)	Median (IQR) (mm)
BD-IPMN	Low-Risk	74 (79.6)	21.9±11.7	18.2 (14.0–29.0)
	High-Risk	19 (20.4)	28.8±15.4	25.1 (15.5–37.5)
MD-IPMN	Low-Risk	33 (42.9)	9.7±5.0	8.6 (6.8–9.9)
	High-Risk	44 (57.1)	11.8±5.8	10.3 (7.9–14.1)

**Table 1 B.** Distribution of Low-Risk and High-Risk IPMN lesions by region and extent categorization in our cohort.

Region	Low-Risk n (%)	High-Risk n (%)
Head	40 (37.4)	12 (19.0)
Body	15 (14.0)	3 (4.8)
Tail	15 (14.0)	8 (12.7)
Multi-regional	37 (34.6)	40 (63.5)
a. Head + Body	12 (11.2)	6 (9.5)
b. Body + Tail	4 (3.7)	8 (12.7)
c. Whole-pancreas	21 (19.6)	26 (41.3)
Extent	Low-Risk n (%)	High-Risk n (%)
Single region	70 (65.4)	23 (36.5)
Two regions	16 (15.0)	14 (22.2)
Whole-pancreas	21 (19.6)	26 (41.3)
Total	107 (100)	63 (100)

**Table 2:** Model accuracy and uncertainty across IPMN characteristics.

Parameter	Group	Accuracy (%)	p-value	Uncertainty (mean ± s.e.m.)	p-value
**Overall**	–	67.1	–	–	–
**Prediction outcome**	Correct	–	–	0.72 (0.01)	**< 0.0001**
	Incorrect	–		0.78 (0.01)	
**Risk**	Low-Risk	81.3	**< 0.001**	0.73 (0.01)	**0.034**
	High-Risk	42.9		0.76 (0.01)	
**Type**	BD-IPMN	71.0	0.254	0.72 (0.01)	**0.012**
	MD-IPMN	62.3		0.76 (0.01)	
**BD size**	<15 mm	81.3	0.164	0.69 (0.02)	**0.048**
	15–30 mm	63.2		0.73 (0.02)	
	> 30 mm	61.5		0.75 (0.02)	
**MD size**	<10 mm	65.9	1.000	0.77 (0.01)	0.461
	≥10 mm	63.6		0.76 (0.01)	
**Region**	Head	69.2	0.074	0.73 (0.01)	0.152
	Body	77.8		0.70 (0.03)	
	Tail	47.8		0.75 (0.02)	
	**Multi-regional**	68.8			
	• Head + Body	66.7		0.73 (0.03)	
	• Body + Tail	41.7		0.77 (0.03)	
	• Whole-pancreas	76.6		0.76 (0.02)	
**Extent**	Single region	65.6	0.168	0.73 (0.01)	0.121
	Two regions	56.7		0.75 (0.02)	
	Whole-pancreas	76.6		0.76 (0.01)	

Note: Uncertainty values represent estimated marginal means ± standard error (s.e.m.) from linear regression models. Reported p-values represent the overall p-value from the ANOVA test.

## Data Availability

Our MRIs and corresponding excel file for risk status of the patients are available at OSF server (NIH supported data sharing platform) at https://osf.io/74vfs/.
